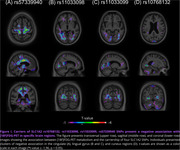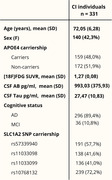# Single nucleotide polimorphisms of the main glutamate transporter in the brain are associated with lower regional brain glucose metabolism in cognitively impaired individuals

**DOI:** 10.1002/alz.094099

**Published:** 2025-01-09

**Authors:** Roberta dos Santos de Oliveira, Christian Limberger, Luiza Santos Machado, Marco Antônio de Bastiani, Thomas Hugentobler Schlickmann, Giovanna Carello‐Collar, Guilherme Povala, Tharick A. Pascoal, Eduardo R. Zimmer

**Affiliations:** ^1^ Universidade Federal do Rio Grande do Sul, Porto Alegre, Rio Grande do Sul Brazil; ^2^ Universidade Federal do Rio Grande do Sul, Porto Alegre, RS Brazil; ^3^ University of Pittsburgh, Pittsburgh, PA USA

## Abstract

**Background:**

Astrocytes play a main role in brain energy metabolism, primarily through the metabolic cooperation with neurons. The use of [18F]fluorodeoxyglucose(FDG)‐PET has become a valuable indicator of neurodegeneration in Alzheimer’s disease (AD), revealing a brain hypometabolic signature, but it is sensitive to changes in astrocyte metabolism. It is postulated that the activation of the excitatory amino acid transporter 2 (EAAT2) is the main trigger of FDG‐PET uptake in astrocytes. However, the potential relationship between single nucleotide polymorphisms (SNPs) in the gene that encodes for EAAT2 (SLC1A2) and the AD hypometabolic signature remains unknown. Here, we investigated whether SNPs in SLC1A2 affect brain glucose metabolism in cognitively impaired (CI) individuals.

**Method:**

We assessed 331 CI individuals from the ADNI cohort with available [18F]FDG‐PET imaging data, CSF Aß1‐42 and p‐tau181 measures, and genotyping data of SLC1A2 (Table 1). After the genetic data processing, 100 SNPs from the SLC1A2 gene were identified. In this study, we focused on the 10 SNPs more associated with [18F]FDG SUVR in an exploratory ROI‐wise analysis. Then, we performed voxel‐wise correlation analysis with RMINC, testing the association between the SLC1A2 SNP carriership and [18F]FDG‐PET, adjusting for age, sex, APOE4 status, and CSF Aß42 and p‐tau181 levels (corrected p‐value < 0.05).

**Result:**

Our findings revealed a negative association between four SLC1A2 SNPs and [18F]FDG‐PET in specific brain regions. This indicates that SLC1A2 SNP carriership is associated with lower regional glucose metabolism. Notably, rs57339940 carriers presented a significant cluster in the cingulate region (tmax = 3.04, p < 0.01, Figure 1A). Carriers of rs11033098 and rs11033099 exhibited bilateral clusters in areas of the lingual gyrus (tmax = 2.4806 and tmax = 2.8736, respectively, p < 0.01, Figure 1B‐C). Additionally, individuals with rs10768132 showed a substantial bilateral impact on the posterior areas of the cuneus (tmax = 2.9821, p < 0.01, Figure 1D).

**Conclusion:**

These preliminary results show that SLC1A2 SNP carriership had a significant influence on regional glucose hypometabolism, specifically in areas comprising the occipital lobe and the cingulate region. Metabolic alterations may influence the susceptibility of those regions to accumulate amyloid in later stages of AD.